# Evaluation of Antiviral Effect against SARS-CoV-2 Propagation by Crude Polysaccharides from Seaweed and Abalone Viscera In Vitro

**DOI:** 10.3390/md20050296

**Published:** 2022-04-27

**Authors:** Sang-Min Kang, Dongseob Tark, Byeong-Min Song, Gun-Hee Lee, Ju-Hee Yang, Hee-Jeong Han, Sung-Kun Yim

**Affiliations:** 1 Laboratory for Infectious Disease Prevention, Korea Zoonosis Research Institute, Jeonbuk National University, Iksan 54531, Korea; sangminkang@jbnu.ac.kr (S.-M.K.); tarkds@jbnu.ac.kr (D.T.); als20079@jbnu.ac.kr (B.-M.S.); ghlee80@jbnu.ac.kr (G.-H.L.); juheeppuing@jbnu.ac.kr (J.-H.Y.); hjeongly@jbnu.ac.kr (H.-J.H.); 2 Marine Biotechnology Research Center, Jeonnam Bioindustry Foundation, 21-7, Nonggongdanji 4Gil, Wando-eup, Wando-gun 59108, Korea

**Keywords:** COVID-19, SARS-CoV-2, seaweed, abalone viscera, polysaccharide, antiviral activity

## Abstract

Crude polysaccharides, extracted from two seaweed species (*Hizikia fusiforme* and *Sargassum horneri*) and *Haliotis discus hannai* (abalone) viscera, were evaluated for their inhibitory effect against SARS-CoV-2 propagation. Plaque titration revealed that these crude polysaccharides efficiently inhibited SARS-CoV-2 propagation with IC_50_ values ranging from 0.35 to 4.37 μg/mL. The crude polysaccharide of *H. fusiforme* showed the strongest antiviral effect, with IC_50_ of 0.35 μg/mL, followed by *S. horneri* and abalone viscera with IC_50_ of 0.56 and 4.37 μg/mL, respectively. In addition, immunofluorescence assay, western blot, and quantitative RT-PCR analysis verified that these polysaccharides could inhibit SARS-CoV-2 replication. In Vero E6 cells, treatment with these crude polysaccharides before or after viral infection strongly inhibited the expression level of SARS-CoV-2 spikes, nucleocapsid proteins, and RNA copies of RNA-dependent RNA-polymerase and nucleocapsid. These results show that these crude marine polysaccharides effectively inhibit SARS-CoV-2 propagation by interference with viral entry.

## 1. Introduction

In the absence of a vaccine or treatment for SARS-CoV-2 after the COVID-19 pandemic, many studies have been conducted across the world to screen substances for inhibition of SARS-CoV-2 infection, replication, and propagation from marine bioactive compounds, such as polyphenols, carotenoids, and polysaccharides [1,2,3,4]. Polyphenols derived from brown algae, commonly known as phlorotannins, were predicted in silico as a potential inhibitor of SARS-CoV-2 3CLpro [5]. Siphonaxanthin, a marine carotenoid extracted from *Codium fragile*, inhibited SARS-CoV-2 pseudovirus cell entry in vitro [6]. In the field of marine compounds, much work has been focused on polysaccharides for the development of therapeutics and prophylaxis for COVID-19.

Recently, it was reported that iota-carrageenan inhibits SARS-CoV-2 replication in various cell lines [7,8] and also inhibits replication of its variants, alpha, beta, gamma, and delta [9]. Lambda-carrageenan has been reported to have antiviral effects against SARS-CoV-2 in vitro [10]. In addition, an iota-carrageenan nasal spray exhibited a relative risk reduction of 79.8% in preventing SARS-CoV-2 in healthcare workers managing patients with COVID-19 disease [11].

Furthermore, a number of marine sulfated polysaccharides (RPI-27, RPI-28, and sulfated galactofucan from *Saccharina japonica*, sea cucumber sulfated polysaccharide (SCSP), and rhamnan sulfate from *Monostroma nitidum*) effectively inhibited SARS-CoV-2 entry by interfering with the interaction of the spike protein with the ACE2 receptor of the host cell in vitro [12,13,14,15,16]. Kwon et al. was reported that RPI-27 (Mw ~ 100 kDa) has a higher molecular weight than RPI-28 (Mw ~ 12 kDa) and is a branched polysaccharide with an EC_50_ value of 8.3 ± 4.6 μg/mL against SARS-CoV-2 compared with RPI-28′s EC_50_ of 16 ± 11 μg/mL [13]. SCSP exhibited strong inhibitory activity in Vero E6 cells with an IC_50_ of 9.10 μg mL^−1^ [15]. In addition, the neutralizing effect of rhamnan sulfate on wildtype and delta SARS-CoV-2 pseudovirus particles in vitro resulted in IC_50_ values of 2.39 and 1.66 μg/mL, respectively [16]. In our previous study [17], the crude polysaccharides (CPs) from seaweed and abalone viscera effectively inhibited SARS-CoV-2 pseudovirus entry. Among them, CPSH (crude polysaccharide from *Sargassum horneri*), CPAV (crude polysaccharide from abalone viscera), and CPHF (crude polysaccharide from *Hizikia fusiforme*) inhibited virus infection with IC_50_ values of 12 μg/mL, 33 μg/mL, and 47 μg/mL, respectively. These marine polysaccharides primarily inhibited SARS-CoV-2 infection in a noncompetitive manner.

In order to show various spectrums of the antiviral activity of marine polysaccharides for application in the development of therapeutics and prophylaxis, we evaluated the inhibitory effect on SARS-CoV-2 propagation in vitro using the three polysaccharides (CPAV, CPSH, and CPHF) that showed the highest inhibition of SARS-CoV-2 pseudovirus cell entry in our previous study [17].

## 2. Results

### 2.1. Cytotoxicity Assay

**Figure 1:**
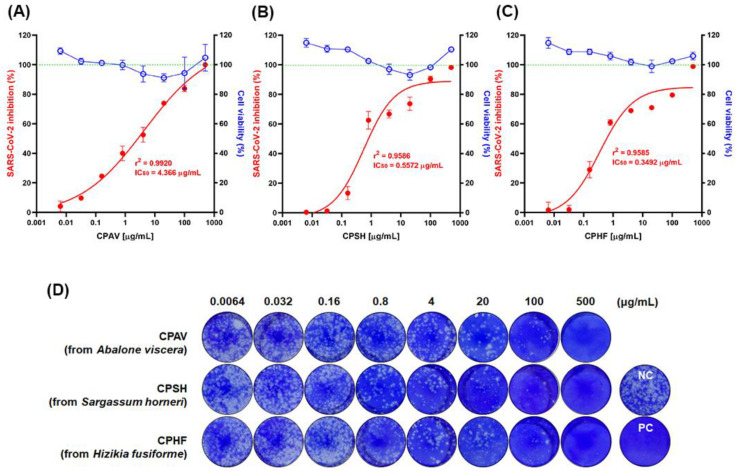
Determination of cellular viability (right *y*-axis, blue open circle) and inhibition of SARS-CoV-2 propagation (left *y*-axis, red closed circle) by crude polysaccharides in Vero E6 cells. Vero E6 cells were treated with various concentrations of (**A**) CPAV (crude polysaccharide from abalone viscera), CPSH (crude polysaccharide from *Sargassum horneri*), and (**C**) CPHF (crude polysaccharide from *Hizikia fusiforme*) from 0.006 μg/mL to 500 μg/mL (5-fold serial dilution) for 72 h at 37 °C, respectively. The cellular viability of Vero E6 cells was assessed using a Cell Titer-Glo 2.0 Luminescent cell viability assay kit (Promega, Madison, WI, USA). (**D**) Plaque reduction assay was performed to evaluate the inhibitory effect on SARS-CoV-2 propagation. Vero E6 cells were pretreated with various concentrations of CPAV, CPSH, CPHF, and remdesivir (1.56~50 μM, 2-fold serial dilutions) as a positive control for 1 h, respectively, and then cells were infected with 1.5 × 10^4^ TCID_50_ of SARS-CoV-2 at an MOI of 0.02 for 1 h. At 3 days post infection (dpi) in the overlay medium, viral plaques were counted in comparison with the mock-treated controls (set at 100%, NC). Counted plaques were expressed as a percent of inhibition (**A**–**C**, left *y*-axis, red closed circle) in crude polysaccharides treated cultures versus untreated and were plotted with GraphPad Prism 8 (GraphPad, San Diego, CA, USA). IC_50_ was calculated by nonlinear regression. Data are expressed as mean ± S.D. of three independent experiments.

For this study, the same crude polysaccharides used in our previous study [17], CPAV, CPSH, and CPHF, were used. The viability of Vero E6 cells was assessed using a Cell Titer-Glo 2.0 reagent (Promega, Madison, WI, USA). The Vero E6 cells were treated with CPHF, CPSH, and CPAV, respectively, at a final concentration range from 0.006 μg/mL to 500 μg/mL (serially diluted 1/5) for 72 h. As a result of treatment with 20 μg/mL of CPs, the cytotoxicity of CPAV, CPSH, and CPHF were 8.87 ± 2.7, 6.93 ± 3.5, and 1.07 ± 4.3%, respectively (Figure 1A–C). The crude polysaccharide from abalone viscera was slightly more toxic than the other two CPs. However, this level of cytotoxicity is not significant enough to offset the antiviral effect.

### 2.2. Quantification of SARS-CoV-2 by Plaque Aassay

A plaque assay was performed to evaluate the antiviral activity of the three crude polysaccharides, CPHF, CPSH, and CPAV, respectively (Figure 1D). After Vero E6 cells were treated with CPs at the final concentration range from 0.006 μg/mL to 500 μg/mL (serially diluted 1/5), the cells were infected with 1.5 × 10^4^ TCID_50_/mL of SARS-CoV-2 virus at a multiplicity of infection (MOI) of 0.02. Remdesivir was used as a positive control at the final concentration range from 1.56 μM to 50 μM (serially diluted 1/2). Based on counting the number of plaques after crystal violet staining, plaque formation was reduced in a dose-dependent manner by CPs and treatment with 500 μg/mL of CPs reduced plaque formation over 98% compared with virus only (without CPs). In addition, plaques were not observed in any cells treated with any concentrations of remdesivir. When Vero E6 cells were treated with 0.8 μg/mL CP concentrations before SARS-CoV-2 infection, plaque formation was reduced over 60% by CPHF and CPSH, but CPAV inhibited plaque formation by only about 40%. The antiviral activity with 50% inhibitory concentration (IC_50_) values of CPs were determined by calculating the virus titer based on the plaque assay (Figure 1A–C). It was observed that propagation of virus mostly decreased with the increasing concentrations in CPs and showed sigmoid graphs. The concentrations with IC_50_ of CPHF, CPSH, and CPAV were 0.35, 0.56, and 4.37 μg/mL, respectively.

### 2.3. TCID_50_ Assay

**Figure 2:**
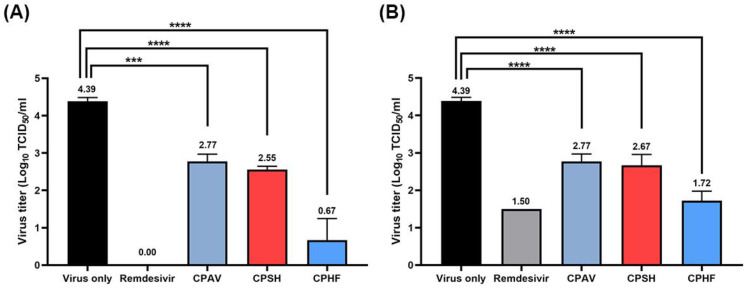
Inhibition of SARS-CoV-2 infection by the crude polysaccharides from abalone viscera (CPAV), *Sargassum horneri* (CPSH), and *Hizikia fusiforme* (CPHF), respectively. Vero E6 cells were treated with 500 μg/mL of CPAV, CPSH, CPHF, and 10 μM remdesivir for 1 h, respectively, and infected with 10-fold serially diluted 1.0 × 10^5^ TCID_50_ of SARS-CoV-2 virus for (**A**) 3 days post infection (dpi) and (**B**) 4 dpi. The fifty percent tissue culture infectious dose (TCID_50_) was determined based on whether or not a cytopathic effect occurred. Data are expressed as the mean ± S.D. of three independent experiments. *** *p* < 0.001 and **** *p* < 0.0001. The graph was created using GraphPad Prism 8 (GraphPad, San Diego, CA, USA).

For determination of 50% tissue culture infectious dose (TCID_50_), Vero E6 cells were treated with 500 μg/mL of CPs, or 10 μM remdesivir (as a positive control), respectively. After SARS-CoV-2 infection, TCID_50_s were determined based on whether or not a cytopathic effect (CPE) occurred at 3 days and 4 days post infection (dpi), respectively. The crude polysaccharides significantly inhibited SARS-CoV-2 infection in Vero E6 cells at 3 dpi and 4 dpi compared with the virus only (Figure 2). However, at 4 dpi, the viral titer was increased in treatments with remdesivir or CPHF, respectively, where it reached a significantly higher titer of 1.50 and 1.72 versus 0 and 0.67 log_10_ TCID_50_/mL (*p* < 0.0001), whereas CPAV and CPSH had no change in the viral titer.

### 2.4. Identification of Antiviral Effects by IFA

**Figure 3:**
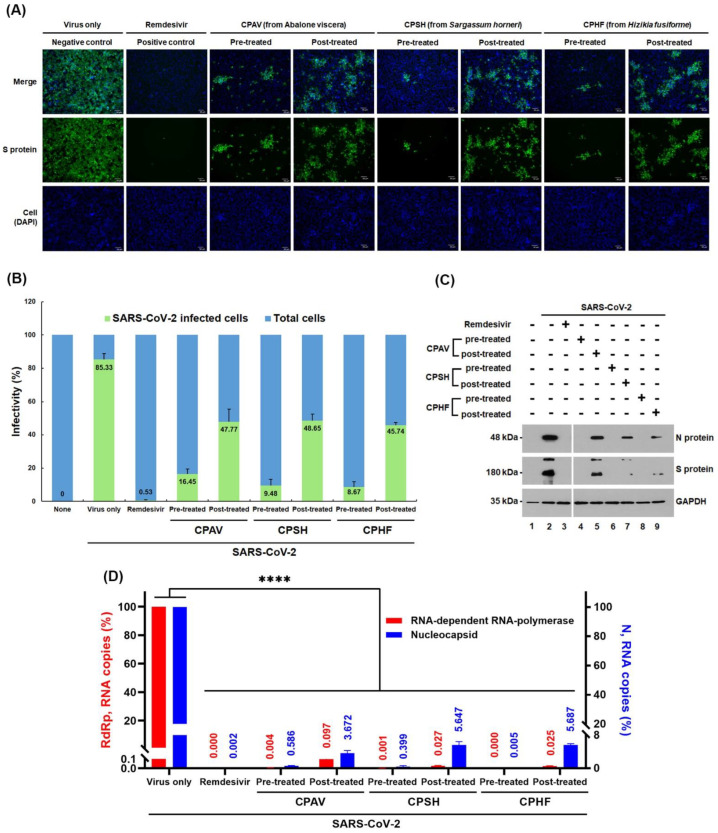
Antiviral effect of the crude polysaccharides (CPs) from abalone viscera (CPAV), *Sargassum horneri* (CPSH), and *Hizikia fusiforme* (CPHF) on the replication of SARS-CoV-2. (**A**) Immunofluorescence assay (IFA). Vero E6 cells were treated with 500 μg/mL of CPs for 1 h before or after cells were infected with 6.0 × 10^4^ TCID_50_ of SARS-CoV-2 virus at an MOI of 0.1 for 1 h, respectively. Remdesivir was used at 10 μM as a positive control and the negative control is nontreated. At 24 h post infection, the SARS-CoV-2 spike protein was detected with a SARS-CoV-2 spike polyclonal antibody (Sino Biological Inc., Beijing, China) and Alexa Fluor 488-conjugated goat anti-mouse IgG (Cell Signaling Technology, Boston, MA, USA) as a secondary antibody (green). Cell nuclei were counterstained with 4′,6-diamidino-2-phenylindole (DAPI, blue). (**B**) Infectivity (%) based on IFA was plotted after calculating the fluorescence intensity of spike protein bound to Alexa Fluor 488-conjugated secondary antibody and the intensity of cell nuclei stained with DAPI. (**C**) Western blot. At 24 h post infection as mentioned above, whole-cell lysates were subjected to immunoblotting with anti-nucleocapsid antibody (upper panel) and SARS-CoV-2 spike antibody (medium panel), in which glyceraldehyde 3-phosphate dehydrogenase (GAPDH) was used as a loading control (lower panel). Proteins are marked on the right side of the panels. None, cell lysates without SARS-CoV-2 infection (lane 1); virus only, SARS-CoV-2-infected cell lysates without CPs (lane 2). N, nucleocapsid; S, spike protein. (**D**) Quantitative RT-PCR. Viral RNA was purified from the cells 24 h post infection as mentioned above. Quantitative RT-PCR was conducted using the SARS-CoV-2 nucleocapsid gene (right *y*-axis, blue) and RNA-dependent RNA-polymerase (RdRp, left *y*-axis, red) gene-specific primers, respectively. Data are expressed as the mean ± S.D. of three independent experiments. **** *p* < 0.0001. The graphs were created using GraphPad Prism 8 (GraphPad, San Diego, CA, USA).

To assess the antiviral effect of CPs on the propagation of SARS-CoV-2 at 24 h post infection, immunofluorescence assay (IFA) was performed using a SARS-CoV-2 spike antibody when Vero E6 cells were treated with 500 μg/mL of CPs, before and after virus infection. There was no fluorescence signal of Alexa fluor 488 in the cells treated with 10 μM of remdesivir (as a positive control) before SARS-CoV-2 infection (Figure 3A), while over 85% of cells were virus-positive in the SARS-CoV-2-infected cells as a negative control (Figure 3B). Infectivity of SARS-CoV-2 based on IFA in the presence of 500 μg/mL of CPAV, CPSH, and CPHF before virus infection was 16.5 ± 2.5%, 9.5 ± 3.2%, and 8.7 ± 2.7%, respectively, while it was increased to 47.8 ± 6.6%, 48.7 ± 3.3%, and 45.7 ± 1.4%, respectively when post-treated with CPs after virus infection. When treated with 10 μM remdesivir before virus infection, the infectivity was 0.53 ± 0.4% (Figure 3B).

### 2.5. Identification of Antiviral Effects by Western Blotting

To examine the expression levels of viral spikes and nucleocapsid proteins, immunoblotting was performed using anti-SARS-CoV-2 nucleocapsid (N) and spike (S) antibodies, respectively, both when Vero E6 cells were pre- or post-treated with 500 μg/mL of CPs. Viral N and S proteins were not detected without virus (Figure 3C, lane 1), while obvious bands of N and S protein were detected at 48 kDa and 180 kDa, respectively, in virus-only treated negative control cells (Figure 3C, lane 2). In order to confirm the suppression of viral protein expression by CPs, when Vero E6 cells were treated with CPAV, CPSH, and CPHF before virus infection, respectively, N and S proteins were not detected (Figure 3C, lane 4, 6, and 8) as with 10 μM remdesivir (positive control, Figure 3C, lane 3). However, N and S proteins were detected in cells treated with CPs after virus infection (Figure 3C, lane 5, 7, and 9). When CPAV was treated, these viral proteins were clearly detected (Figure 3C, lane 5) although the amount of detection was small compared with virus only. In addition, when CPSH and CPHF were used after viral infection, the expression level of the spike protein was significantly reduced compared with that of nucleocapsid.

### 2.6. Identification of Antiviral Effects by qRT-PCR

To examine the inhibitory effect of CPs on SARS-CoV-2 replication, quantitative RT-PCR (qRT-PCR) was performed using RNA-dependent RNA polymerase (RdRp) and nucleocapsid (N) primers (Table 1), respectively. Vero E6 cells were treated with 500 μg/mL of CPs before or after virus infection for 24 h post infection (Figure 3D). As a result, RdRp and N RNA copies were not detected in Vero E6 cells pretreated with 10 μM remdesivir as a positive control (0.000 ± 0.00001% against RdRp and 0.001 ± 0.0003% against nucleocapsid), CPAV (0.004 ± 0.001% against RdRp and 0.586 ± 0.093% against nucleocapsid), CPSH (0.001 ± 0.0004% against RdRp and 0.399 ± 0.247% against nucleocapsid), and CPHF (0.000 ± 0.00002% against RdRp and 0.005 ± 0.002% against nucleocapsid) when compared with SARS-CoV-2-infected cells without treatment (Figure 3D). However, when Vero E6 cells were post-treated with CPs after SARS-CoV-2 infection, RdRp and N RNA copies were slightly increased at 0.097 ± 0.003%, 0.027 ± 0.005%, and 0.025 ± 0.002% against RdRp and 3.672 ± 0.649%, 5.647 ± 0.850%, and 5.687 ± 0.313% against nucleocapsid, respectively.

### 2.7. Reporter Assay Using SARS-CoV-2 Pseudovirus

**Figure 4:**
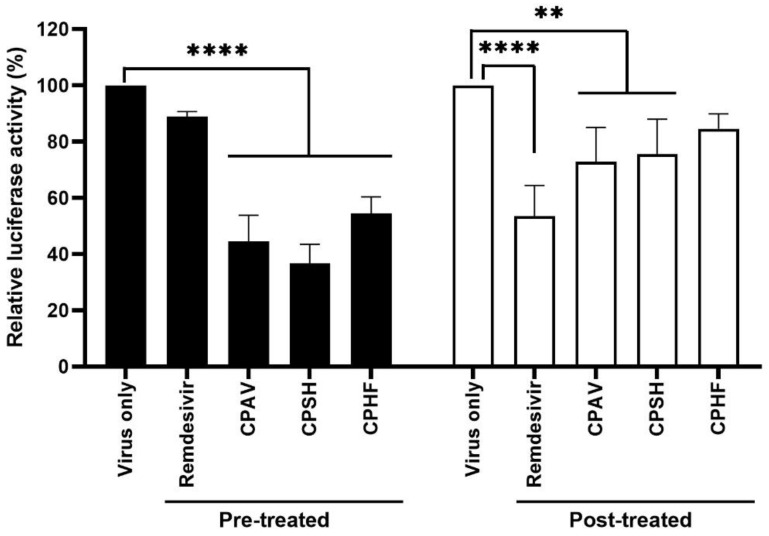
Inhibition of SARS-CoV-2 pseudovirus cell entry. Lentiviral pseudotypes bearing SARS-CoV-2 spike protein were prepared in which a firefly luciferase-expressing plasmid was incorporated. HEK293T cells were treated with 500 μg/mL of crude polysaccharides and 10 μM remdesivir for 1 h before or after cells were infected with the SARS-CoV-2 pseudovirus (about 10,000 luminescence) for 4 h, respectively. At 24 h post infection, relative luciferase activity was determined by fixing the nontreated sample at 100%. Data are expressed as the mean ± S.D. of two independent experiments. ** *p* < 0.01 and **** *p* < 0.0001. The graph was created using GraphPad Prism 8 (GraphPad, San Diego, CA, USA).

To investigate whether the antiviral effects of CPs were due to the inhibition of SARS-CoV-2 entry or the inhibition of viral replication, a SARS-CoV-2 pseudovirus was prepared, and HEK293T cells were treated with 500 μg/mL of CPs or 10 μM remdesivir before or after the SARS-CoV-2 pseudovirus infection, respectively (Figure 4). When pretreating with CPs before pseudovirus infection, SARS-CoV-2 pseudovirus cell entry was strongly inhibited, but the inhibitory effect was relatively low when post-treating with CPs.

## 3. Discussion

A number of marine polysaccharides, comprised of carrageenan, laminarin, alginate, fucoidan, chitosan, ulvan, agar, and porphyran, have exhibited directly and indirectly antiviral effects against SARS-CoV-2 [7,8,9,10,11,12,13,14,15,16]. Iota-carrageenan inhibits the replication of SARS-CoV-2 and its variants alpha, beta, gamma, and delta [9], and lambda-carrageenan also has demonstrated antiviral activity against influenza virus and SARS-CoV-2 [7,8]. Sulfated polysaccharides effectively inhibited SARS-CoV-2 entry by interfering with the interaction of the spike protein with the ACE2 receptor of the host cell in vitro [13,14,15,16]. In our previous study [17], crude polysaccharides (CPs) from seaweed and abalone viscera inhibited SARS-CoV-2 pseudovirus cell entry with an IC_50_ of 12~289 μg/mL. Among them, the total carbohydrate content was the highest in CPSH (99.1%), followed by CPHF (94.4%) and CPAV (62.7%), whereas the highest level of total protein content was found in CPAV (22.3%), followed by CPHF (10.9%) and CPSH (4.0%), respectively. In addition, CPHF had the highest sulfate ion content (20.4%), followed by CPSH (9.8%) and CPAV (0.5%), respectively. In monosaccharide composition, total fucose and galactose contents were CPSH (66.2% and 6.9%), CPHF (41.8% and 9.6%), and CPAV (37.3% and 12.9%), respectively [17].

In this study, the same crude polysaccharides used in our previous study [17], CPAV, CPSH, and CPHF, which effectively inhibited SARS-CoV-2 pseudovirus entry in that study, were evaluated for antiviral effects against SARS-CoV-2 (NCCP 43326/Wuhan) propagation in vitro. CPAV, CPSH, and CPHF drastically inhibited SARS-CoV-2 propagation in a dose-dependent manner (Figure 1). As described previously for crude polysaccharides [17], these results suggest that the antiviral effect of CPs could mainly be explained by interference with SARS-CoV-2 entry because propagation of SARS-CoV-2 was strongly inhibited and the virus titer sharply reduced when CPs were used to pretreat before viral infection (Figure 1 and Figure 2), while the inhibitory effect was less significant when CPs were used for post-treatment after viral infection (Figure 3 and Figure 4). These results show that, although not purified polysaccharides, CPs can inhibit SARS-CoV-2 propagation as well as cell entry, and high molecular weight, high total carbohydrate, and high fucose content are important factors in the inhibitory effect. Kwon et al. [13] reported that RPI-27(~100 kDa), a branched polysaccharide from *Saccharina japonica*, was shown to have higher antiviral activity (EC_50_ = 8.3 μg/mL) than RPI-28 (~12 kDa, EC_50_ = 16 μg/mL). Jin et al. [14] reported that sulfated galactofucan (195 kDa) and glucuronomannan (7.0 kDa) strongly inhibited interaction between the SARS-CoV-2 S-glycoprotein and heparin (IC_50_s are 27 and 231 nM, respectively). In addition, CPAV has a higher total protein content compared with CPSH and CPHF, which is presumed to exhibit strong antiviral activity due to the synergistic effect of various bioactive molecules [18,19,20,21] and high molecular weight polysaccharides present in the abalone viscera.

Furthermore, the crude polysaccharides clearly inhibited SARS-CoV-2 replication and viral protein expression even as a post-treatment. These results are supported by IFA, western blot, and qRT-PCR (Figure 3). The inhibition of virus replication and viral protein expression by CPs may be due to the post-treatment CPs inhibiting the re-infection of surrounding cells by released virions after replication. These results show a more significant inhibitory action with the prophylactic application. In the immunofluorescence assays (IFA), the CP pretreatment blocked about 90% of SARS-CoV-2 propagation, although CPAV displayed a lower inhibitory effect than CPSH and CPHF, and CP post-treatment blocked about 60% (Figure 3A). Immunoblotting supported that the pretreatment inhibited the expression of SARS-CoV-2 spikes and nucleocapsid proteins, but the post-treatment did not completely inhibit it (Figure 3C). In addition, pre and post-treatment with CPs dramatically reduced RdRp and nucleocapsid RNA copies similar to remdesivir as a positive control in immunofluorescence assays (Figure 3D). Similar to these results, testing with a SARS-CoV-2 pseudovirus for cell entry showed a high inhibitory effect when CPs were administered before virus infection compared with after virus infection (Figure 4). It is presumed that crude polysaccharides from seaweed and abalone viscera inhibit SARS-CoV-2 cell entry, resulting in the inhibition of viral RNA replication and protein expression. Fröba et al. and Varese et al. reported that iota-carrageenan inhibits the replication of SARS-CoV-2 in various cell lines [8,9], and iota-carrageenan also inhibited the variants alpha, beta, gamma, and delta [9].

There was a slight correlation between molecular weight of the CPs and antiviral inhibitory effects. Regarding the IC_50_ of CPs, the antiviral activity of CPAV increased in a dose-dependent manner, while CPSH and CPHF showed almost no inhibitory effect at concentrations below 0.032 μg/mL. However, these showed strong antiviral effects at concentrations above 0.16 μg/mL and the effects increased gently at concentrations above 0.8 μg/mL. When CPSH and CPHF were used at concentrations below 0.032 μg/mL, plaques formed 98.8 ± 1.1% and 97.9 ± 2.9%, respectively, and with CPAV they formed 90.2 ± 0.1%. On the other hand, when CPSH and CPHF were used at concentrations over 0.8 μg/mL, plaque formation was sharply reduced to 37.5 ± 5.1% and 39.0 ± 2.8% compared with CPAV (59.9 ± 3.7%). In addition, the plaque formation with CPs was similarly reduced to 26.1 ± 0.8% (CPAV), 26.3 ± 3.9% (CPSH), and 29.1 ± 1.7% (CPHF), respectively, when CPs were used at concentrations over 20 μg/mL. These differences seem to be related to the difference in molecular weight of the CPs. In our previous study, CPAV appeared to be constituted of whole polysaccharides and fragments (over 800 kDa and 400~800 kDa) due to degradation by marine microorganisms in the abalone viscera, whereas CPSH and CPHF were both over 800 kDa [17]. We suspect that the small molecular weight of CPAV nonspecifically interferes with the binding of the SARS-CoV-2 spike with the ACE receptor at low concentrations (Figure 1A), but large molecular weight CPs inhibit it more effectively at higher concentrations (Figure 1B,C). These results show that crude polysaccharides of over 800 kDa required at least a certain concentration of CPs to effectively inhibit SARS-CoV-2 cell entry.

## 4. Materials and Methods

### 4.1. Chemicals and Reagents

Dulbecco’s modified Eagle’s medium (DMEM), fetal bovine serum (FBS), phosphate buffered saline (PBS) pH 7.4, and geneticin (G-418 sulfate) were purchased from Thermo Fisher Scientific (Waltham, MA, USA), and the Cell Titer-Glo Luminescent cell viability assay kit was purchased from Promega (Madison, WI, USA). Dimethyl sulfoxide (DMSO), agarose, formaldehyde solution (37%), chloroform, 2-propanol, and crystal violet were purchased from Sigma-Aldrich (St. Louis, MO, USA).

CPAV (from abalone viscera), CPSH (from *Sargassum horneri*), and CPHF (from *Hizikia fusiforme*) were prepared for use in our previous study. The algae *Hizikia fusiforme* (collected in May 2020), *Sargassum horneri* (collected in August 2020), and abalone (*Haliotis discus hannai*, Dashimachonbok) were cultured in Wando, Jeollanam-do, South Korea. The fresh seaweed and abalone viscera were immediately washed with tap water and then dried. A concentrated water extract was rendered from each by treating with 2% CaCl_2_ and 1.2 L of 85% (*v/v*) ethanol to remove alginic acid and dialyzed using an ultrafiltration hollow fiber cartridge (10,000 NMWC, 31.8 L × 3.2 cm O.D., Cytiva, Middlesex County, MA, USA). Finally, the CP was lyophilized and stored at −20 °C [17].

### 4.2. Cells and Viruses

African green monkey kidney Vero E6 cell culture (ATCC CRL-1586^TM^) was purchased from the American Type Culture Collection (Manassas, VA, USA). Vero E6 cells were maintained at 37 °C, 5% CO_2_ in Dulbecco’s modified Eagle’s medium (DMEM; HyClone) supplemented with 10% FBS. SARS-CoV-2 (NCCP 43326/Wuhan) virus was provided by the Korea Disease Control and Prevention Agency and was amplified in Vero E6 cells at 37 °C for 3 days. After centrifugation at 1000× *g* for 5 min, viral stocks were stored at −80 °C and viral titers were determined in a plaque reduction assay. Vero E6 cells were seeded into 96-well microplates and grown overnight at 37 °C under 5% CO_2_. Ten-fold serial dilutions of SARS-CoV-2 virus from Vero E6 cells were added to monolayers of 80% confluent Vero E6 cells at 37 °C for 1 h in serum-free DMEM. For titer determination of SARS-CoV-2 virus stocks, Vero E6 cells were infected with serial dilutions of the virus stock over 72 h.

### 4.3. Cytotoxicity Assay

The cytotoxicity evaluation of CPHF, CPSH, and CPAV were performed using a Cell Titer-Glo Luminescent cell viability assay kit. Vero E6 cells were seeded into 96-well plates at a density of 2 × 10^4^ cells per well for 24 h at 37 °C under 5% CO_2_. The supernatants were then removed and washed one time with PBS. After the Vero E6 cells were treated with 20 μL of CPHF, CPSH or CPAV at various concentrations (5-fold serial dilutions in the range of 0.006 μg/mL to 500 μg/mL, final D.W 5%) for 72 h, respectively, 100 μL of Cell Titer-Glo 2.0 reagent was added per well and the plate was shaken for 2 min at 700 rpm. After 10 min, the mixture was read via luminometer and cell viability was calculated as follows:

(1) $$\mathrm{Cell}\;\mathrm{Viability}\;\left(\%\right)=\frac{\mathrm{RLU}\;\mathrm{sample}}{\mathrm{RLU}\;\mathrm{conc}.}\times 100$$

where the RLU sample is the luminescence of the experimental sample and RLU conc. is the luminescence of the control. Cytotoxicity was calculated as follows:

(2) $$\mathrm{Cytotoxicity}\;\left(\%\right)=100-\%\mathrm{Cell}\;\mathrm{Viability}$$

The 50% cytotoxic concentration for CPHF, CPSH, and CPAV were not determined because there is no cytotoxicity. Each sample was analyzed in triplicate wells and repeated three times.

### 4.4. Plaque Reduction Assay

The plaque reduction assay was performed as described previously, with some modification [22]. Various concentrations of CPHF, CPSH, and CPAV were prepared as 5-fold serial dilutions in the range from 0.006 μg/mL to 500 μg/mL using distilled water. Remdesivir was prepared using DMSO at various concentrations in the range from 1.56 μM to 50 μM as a positive control. Vero E6 cells were seeded into 12-well plates at a density of 3 × 10^5^ cells per well for 24 h at 37 °C under 5% CO_2_. Cells were treated with CPs for 1 h and infected with 1.5 × 10^4^ TCID_50_ of SARS-CoV-2 virus at an MOI of 0.02 for 1 h. Then, cells were overlaid with a final 0.6% agarose solution and incubated at 37 °C. At 3 days post-infection; cells were fixed with 3.65% formaldehyde for 1 h at room temperature and washed three times with PBS. Finally, the number of plaques was counted by 0.5% crystal violet staining. Each sample was analyzed in duplicate.

### 4.5. TCID_50_ Assay

For determination of 50% tissue culture infectious dose (TCID_50_), Vero E6 cells were seeded into 96-well plates at a density of 2.0 × 10^4^ cells per well for 24 h at 37 °C under 5% CO_2_. Cells were treated with 500 μg/mL of CPs and 10 μM remdesivir for 1 h and infected with 10-fold serially diluted 1.0 × 10^5^ TCID_50_ of SARS-CoV-2 virus for 3 days post infection (dpi) and 4 dpi. TCID_50_s were determined based on whether or not a cytopathic effect occurred. The infective dose 50 was then calculated by the method of Reed and Muench [23]. Each sample was analyzed in triplicate wells and repeated three times.

### 4.6. Immunofluorescence Assay

To assess the propagation of SARS-CoV-2 when Vero E6 cells were treated with 500 μg/mL of CPs before or after virus infection, immunofluorescence assays (IFA) were performed using a SARS-CoV-2 spike antibody. Vero E6 cells were seeded into 12-well plates at a density of 3 × 10^5^ cells per well for 24 h at 37 °C under 5% CO_2_. Cells were treated with CPs for 1 h before or after infection with 6.0 × 10^4^ TCID_50_ of SARS-CoV-2 virus at an MOI of 0.1 for 1 h, respectively. Remdesivir was used at 10 μM as a positive control. At 24 h post infection, the supernatants were removed and washed three times with PBS. Washed cells were fixed with 4% paraformaldehyde and permeabilized with 0.1% Triton X-100. Then cells were washed three times with PBS and blocked with 5% bovine serum albumin (BSA, Bovogen Biologicals, Keilor East, VIC, Australia) for 3 h. Cells were incubated with a SARS-CoV-2 spike polyclonal antibody diluted 1:5000 (Sino Biological Inc., Beijing, China) for 90 min. After washing three times with PBS, the anti-spike antibody bound cells were subsequently labeled for 1 h at room temperature with Alexa Fluor 488-conjugated goat anti-mouse IgG diluted 1:2000 (Cell Signaling Technology, Boston, MA, USA), and the nuclei were stained with 4′,6-diamidino-2-phenylindole diluted 1:2000 (DAPI, Thermo Fisher Scientific, Waltham, MA, USA) for 30 min. Confocal laser scanning microscopy was performed with a CELENA^®^ S Digital Imaging System (Logos Biosystems, Anyang, South Korea). Each sample was analyzed in triplicate.

### 4.7. Western Blotting

To examine the expression of viral spike and nucleocapsid proteins, immunoblotting was performed using anti-SARS-CoV-2 spike (S) and nucleocapsid (N) antibodies, both when Vero E6 cells were treated with 500 μg/mL of CPs for 1 h before or after cells were infected with 6.0 × 10^4^ TCID_50_ of SARS-CoV-2 virus at an MOI of 0.1 for 1 h, respectively. Remdesivir was used at 10 μM as a positive control. At 24 h post infection, the supernatants were removed and washed three times with PBS. Washed cells were lysed by treatment with RIPA lysis buffer (Seongnam-si, Gyeonggi-do, South Korea), and the lysed cells were then centrifuged at 4 °C, 15,000× *g*, for 15 min. Equal amounts of each virus-infected cell lysate or each virus lysate were resolved in 4 × Laemmli sample buffer (Bio-Rad, Hercules, CA, USA) and boiled at 100 °C for 10 min. After gel electrophoresis, the proteins were transferred onto a PVDF membrane which was blocked with PBS containing 5% BSA for 2 h at room temperature. Then, the membrane was incubated with primary antibodies specific to SARS-CoV-2 spike (1 μg) and nucleocapsid proteins (1 μg, Sino Biological Inc., Beijing, China) in PBST at 4 °C for 16 h, followed by washing six times with TBS-T. The washed membrane was incubated in PBST containing horseradish peroxidase-conjugated secondary antibody against rabbit IgG (1 μg, Cell Signaling Technology, Boston, MA, USA) for 1 h at room temperature and then visualized with ECL solution (ELPIS-BIOTECH, Daejeon, South Korea).

### 4.8. RNA Isolation and Quantitative RT-PCR

To quantify SARS-CoV-2 RNA copies, quantitative RT-PCR (qRT-PCR) was performed using RNA-dependent RNA polymerase (RdRp) and nucleocapsid (N) primers (Table 1), respectively. Vero E6 cells were seeded into 12-well plates at a density of 3 × 10^5^ cells per well for 24 h at 37 °C under 5% CO_2_ and treated with 500 μg/mL of CPs for 1 h before or after cells were infected with 6.0 × 10^4^ TCID_50_ of SARS-CoV-2 virus at an MOI of 0.1 for 1 h, respectively. Remdesivir was used at 10 μM as a positive control. At 24 h post infection, the supernatants were removed and washed three times with PBS. The cells were then lysed by treatment with 0.5 mL of TriZol (Invitrogen, Waltham, MA, USA), followed by adding 0.1 mL of chloroform. After centrifugation at 13,000 rpm for 10 min, the supernatant was treated with 0.25 mL of 2-propanol at 4 °C for 10 min, and RNA was then precipitated by centrifugation at 13,000 rpm for 15 min. The precipitated RNA was treated with 0.5 mL of 75% ethanol and centrifuged at 7000 rpm for 5 min to remove ethanol and then dried for 10 min. RNA was suspended by treatment with RNase-free water at 55 °C for 10 min, then RNA was quantified at 100 ng/μL and cDNA was synthesized using All-in-One 5X cDNA Master Mix (Cellsafe, Yongin, South Korea). The PCR reaction was performed at 25 °C for 5 min, 42 °C for 1 h, and 85 °C for 5 s according to the manufacturer’s instructions. Quantitative real-time PCR (qRT-PCR) was performed using the synthesized cDNA as a template at 95 °C for 10 s and at 55 °C for 30 s for 40 cycles with iQ SYBR Green (Bio-Rad, Hercules, CA, USA). PCR primers were used as follows: Actin_fwd: 5′-TGA-CAG-CAG-TCG-GTT-GGA-GCG-3′, Actin_rev: 5′-GAC-TTC-CTG-TAA-CAA-CGC-ATC-TCA-TA-3′, Nucleocapsid_fwd: 5′-TAA-TCA-GAC-AAG-GAA-CTG-ATT-A-3′, Nucleocapsid_rev: 5′-CGA-AGG-TGT-GAC-TTC-CAT-G-3′, RdRp_fwd: 5′-AGA-ATA-GAG-CTC-GCA-CCG-TAG -3′, and RdRp_rev: 5′- CTCCTCTAGTGGCGGCTATT-3′. Each sample was analyzed in triplicate.

### 4.9. Reporter Assay Using SARS-CoV-2 Pseudovirus

For preparing the SARS-CoV-2 pseudovirus, HEK293T cells were treated with 2 μg of pLV-Luc, psPAX2, and pAGCN-SARS-CoV-2, respectively, and 12 μL of lipofectamine 2000 (Thermo Fisher, Waltham, MA, USA) and incubated at 37 °C under 5% CO_2_ for 3 days. After centrifugation at 1000 rpm, at 4 °C for 5 min, the supernatant was treated with Lenti-X concentrator (TaKaRa Bio inc., San Jose, CA, USA) in a ratio of 1:3, and left at 4 °C for 16 h. The supernatant was removed after centrifugation at 1500× *g*, 4 °C for 45 min and the pellet was suspended in serum-free DMEM and stored at −80 °C until use. For the pseudovirus efficiency test, HEK293T cells were infected with the prepared SARS-CoV-2 pseudovirus, and 24 h later the cells were lysed with reporter lysis buffer (Promega, Madison, WI, USA). The lysate was dispensed in a 96-well white plate, the same amount of Bright-Glo reagent (Promega, Madison, WI, USA) was added, and the luminescence was measured with a luminometer (GM3000, Promega, Madison, WI, USA) to confirm the infection efficacy of the pseudovirus. To investigate the inhibitory effect of crude polysaccharides before viral infection, HEK293T cells were treated with 500 μg/mL each of CPHF, CPSH, and CPAV, incubated at 37 °C for 1 h, and then infected with 130 μL of SARS-CoV-2 pseudovirus (about 10,000 luminescence) for 4 h. At the same time, to investigate the inhibitory effect of crude polysaccharides after viral infection, HEK293T cells were infected with 130 μL of SARS-CoV-2 pseudovirus (about 10,000 luminescence) for 4 h, and then treated with 500 μg/mL each of CPHF, CPSH, and CPAV for 1 h at 37 °C. The medium was replaced with DMEM containing 5% FBS, and after 24 h the cells were lysed by treatment with reporter lysis buffer. Lysates were dispensed in a 96-well white plate, the same amount of Bright-Glo reagent was added, and then luminescence was measured. Each sample was analyzed in duplicate wells and repeated two times.

### 4.10. Statistical Analysis

The half maximal inhibitory concentrations (IC_50_) were calculated using nonlinear regression analysis of GraphPad Prism version 8 by plotting (inhibitor) versus response (variable slope, four parameters). The equation corresponds to:

(3) $$Y=\mathrm{Bottom}+\left(\mathrm{Top}-\mathrm{Bottom}\right)/\left(1+{\left(\mathrm{IC}50/X\right)}^{\mathrm{HillSlope}}\right)$$

where Y is the response, X is the concentrations or doses, Top and Bottom are plateaus in the units of the Y axis, and HillSlope describes the steepness of the family of curves. Statistical analyses were performed by ordinary one-way ANOVA *t*-test according to the Dunnett’s multiple comparison method using GraphPad Prism version 8. *p* values lower than 0.05 were considered statistically significant. * *p* < 0.05; ** *p* < 0.01; *** *p* < 0.001; **** *p* < 0.0001.

## 5. Conclusions

This study confirms that the crude polysaccharides of seaweed and abalone viscera can inhibit SARS-CoV-2 propagation before and after viral infection and has shown that they completely inhibit replication through preventing viral entry in vitro. However, these crude polysaccharides must be further purified into substances with high antiviral activity which may be characterized in order to be used in the development of therapeutics and prophylactics against viral spread in settings without vaccines or other therapeutics. Additional investigations are needed to perform nonclinical trials, examine the pharmacokinetics, and clarify the antiviral mechanisms once the structure of the marine polysaccharides is analyzed.

## Figures and Tables

**Table 1:** Primer sequences for detection of SARS-CoV-2 RNA.

Gene Name	Primer Sequences (5’ → 3’)	
Actin	Forward	TGACAGCAGTCGGTTGGAGCG
	Reverse	GACTTCCTGTAACAACGCATCTCATA
Nucleocapsid	Forward	TAATCAGACAAGGAACTGATTA
	Reverse	CGAAGGTGTGACTTCCATG
RdRp	Forward	AGAATAGAGCTCGCACCGTAG
	Reverse	CTCCTCTAGTGGCGGCTATT

## Data Availability

The data presented in this study are available on request from the corresponding author.

## Abbreviations

CP: crude polysaccharide; CPHF: crude polysaccharide from *Hizikia fusiforme*; CPSH: crude polysaccharide from *Sargassum horneri*; CPAV: crude polysaccharide from abalone viscera; IFA: Immunofluorescence assay.
